# Performance evaluation of the high-throughput quantitative Alinity m BK virus assay

**DOI:** 10.1128/jcm.01354-23

**Published:** 2024-03-25

**Authors:** Julie W. Hirschhorn, Mark M. Sasaki, April Kegl, Tanjina Akter, Tanisha Dickerson, Momka Narlieva, Nhi Nhan, Tianxi Liu, Patricia Jim, Stephen Young, Erika Orner, Phyu Thwe, Danijela Lucic, D. Yitzchak Goldstein

**Affiliations:** 1 Department of Pathology and Laboratory Medicine, Medical University of South Carolina, Charleston, South Carolina, USA; 2 Molecular Diagnostics of Abbott, Des Plaines, Illinois, USA; 3 Department of Pathology, Montefiore Medical Center, Bronx, New York, USA; 4 TriCore Reference Laboratories, Albuquerque, New Mexico, USA; St. Jude Children's Research Hospital, Memphis, Tennessee, USA

**Keywords:** nucleic acid amplification test, transplantation, BKV, viral load monitoring, high-throughput diagnostics

## Abstract

BK virus (BKV) infection or reactivation in immunocompromised individuals can lead to adverse health consequences including BKV-associated nephropathy (BKVAN) in kidney transplant patients and BKV-associated hemorrhagic cystitis (BKV-HC) in allogeneic hematopoietic stem cell transplant recipients. Monitoring BKV viral load plays an important role in post-transplant patient care. This study evaluates the performance of the Alinity m BKV Investigational Use Only (IUO) assay. The linearity of the Alinity m BKV IUO assay had a correlation coefficient of 1.000 and precision of SD ≤ 0.25 Log IU/mL for all panel members tested (2.0–7.3 Log IU/mL). Detection rate at 50 IU/mL was 100%. Clinical plasma specimens tested comparing Alinity m BKV IUO to ELITech MGB Alert BKV lab-developed test (LDT) on the Abbott *m*2000 platform using specimen extraction protocols for DNA or total nucleic acid (TNA) resulted in coefficient of correlation of 0.900 and 0.963, respectively, and mean bias of 0.03 and −0.54 Log IU/mL, respectively. Alinity m BKV IUO compared with Altona RealStar BKV and Roche cobas BKV assays demonstrated coefficient of correlation of 0.941 and 0.980, respectively, and mean bias of −0.47 and −0.31 Log IU/mL, respectively. Urine specimens tested on Alintiy m BKV IUO and ELITech BKV LDT using TNA specimen extraction had a coefficient of correlation of 0.917 and mean bias of 0.29 Log IU/mL. The Alinity m BKV IUO assay was performed with high precision across the dynamic range and correlated well with other available BKV assays.

**IMPORTANCE:**

BK virus (BKV) in transplant patients can lead to adverse health consequences. Viral load monitoring is important in post-transplant patient care. This study evaluates the Alinity m BKV assay with currently available assays.

## INTRODUCTION

BK virus (BKV) is a small double-stranded DNA virus in the Polyomaviridae family with seroprevalence of over 90% in the adult population (1, 2). After primary infection, BKV remains latent in tubular epithelial cells and uroepithelial cells. In immunocompetent individuals, BKV is controlled through an adaptive immune response that normally develops soon after initial exposure to viral antigens most likely dependent on the induction of stable antiviral memory T-cell response, similar to other persistent viral infections (3, 4). Immunocompromised individuals such as transplant recipients are at risk of complications due to BKV reactivation or new infections from BKV-positive donors. Up to 10% of kidney transplant recipients develop BKV-associated nephropathy (BKVAN) with a majority of BKVAN occurring within the first year post-transplant (5). BKV-associated hequantiativemorrhagic cystitis (BKV-HC) occurs in up to 25% of pediatric and 54% of adult recipients of allogeneic hematopoietic stem cell transplant (HSCT) between 2 and 8 weeks post-transplantation (6).

Plasma BKV viral loads >4 Log copies/mL are associated with increased rate of biopsy-proven BKVAN (7). BKVAN is considered probable in kidney transplant patients with sustained viremia above 3 Log copies/mL, and diagnosis of presumptive nephropathy is made in viremia above 4 Log copies/mL (7, 8). Diagnosis of BKV-HC requires (i) clinical symptoms/signs of cystitis, (ii) hematuria Grade 2 or higher, and (iii) BKV viruria >7 Log copies/mL (6). In allogeneic HSCT recipients, BKV viremia of >3–4 Log copies/mL is observed in more than two-thirds of patients, and declining viremia viral load was found to correlate with clinical recovery (6).

In the United States, majority of labs are using lab developed tests (LDTs), analyte-specific reagents (ASRs) run as LDTs (e.g., ELITech BKV, Altona BKV, and QIAGEN BKV), or an IVD assay, which currently only includes the Roche cobas BKV assay. The Alinity m BKV IUO assay, when approved by the Food and Drug Administration (FDA), would provide another IVD assay for BKV testing. In this multisite study, we evaluated the analytical and clinical performance of the quantitative qPCR Alinity m BKV IUO assay and compared it to the ELITech MGB Alert BKV assay, the Altona RealStar BKV assay, and the Roche cobas BKV assay.

## MATERIALS AND METHODS

### Study design and specimens

The performance of the Alinity m BKV IUO assay for plasma specimens was compared to two ASR assays, ELITech MGB Alert BKV and Altona RealStar BKV assays run as LDTs, and one IVD assay, the Roche cobas BKV assay. ELITech MGB Alert BKV LDT was run on the Abbott *m*2000*sp/rt* (Abbott Laboratories, Des Plaines, IL, USA) as the test of record (TOR) at Montefiore Medical Center (Bronx, NY, USA) and the Medical University of South Carolina (MUSC) Department of Pathology and Laboratory Medicine (Charleston, SC, USA). Montefiore Medical Center utilized a DNA sample extraction protocol, whereas MUSC utilized a total nucleic acid (TNA) sample extraction protocol on the *m*2000*sp*. ELITech MGB Alert BKV results were obtained at Montefiore Medical Center and MUSC through routine clinical specimen testing. The specimens were de-identified prior to testing with Alinity m BKV IUO at the two sites. Plasma specimens were tested at Montefiore Medical Center and MUSC and urine specimens were tested at MUSC.

De-identified plasma specimens were obtained from iProcess Global Research (Irving, TX, USA) with TOR results from the Altona RealStar BKV ASR assay. Specimens from iProcess were stored at −70°C after receipt at Molecular Diagnostics of Abbott (Des Plaines, IL), specimen aliquots were prepared with one aliquot shipped to TriCore Reference Laboratories (Albuquerque, NM, USA) for Roche cobas BKV testing and a second aliquot prepared for testing with Alinity m BKV IUO at Molecular Diagnostics of Abbott.

At MUSC, the study protocol was considered a quality improvement project and was not subject to institutional review board (IRB) review per MUSC operating procedure. Testing conducted at Molecular Diagnostics of Abbott and TriCore Reference Laboratories was approved by WCG IRB. The study protocol for testing conducted at Montefiore Medical Center was approved by an internal IRB. The study was performed in accordance with the principles of Good Clinical Practice and conducted in adherence with the Declaration of Helsinki.

### Molecular assays

The Alinity m BKV IUO assay is run on the Alinity m analyzer, a high-throughput fully automated analyzer with continuous and random-access capabilities. The Alinity m BKV IUO assay is a dual-target real-time PCR assay targeting conserved regions within the VP2/3 and small t-antigen in the BKV genome. The Alinity m BKV IUO assay has a sample input volume of 500 µL and a quantitative range of 1.70–9.00 Log IU/mL for plasma and urine specimens (9).

The ELITech MGB Alert BKV LDT is a real-time PCR test targeting the VP1 gene run on the *m*2000 platform, a batch analyzer that includes automated specimen preparation on the *m*2000*sp* and PCR on *m*2000*rt*. The ELITech BKV LDT has a sample input volume of 500 µL and a quantitative range between 2.30–7.30 Log IU/mL for plasma specimens and 4.30–7.30 Log IU/mL for urine specimens (10) . Specimen preparation on the *m*2000*sp* at Montefiore Medical Center utilized DNA-specific extraction, whereas specimen preparation on the *m*2000*sp* at MUSC utilized TNA-specific extraction with the same specimen input volume.

Altona RealStar BKV assay (Altona Diagnostics GmbH, Hamburg, Germany) is a real-time PCR assay with quantitative range between 0 Log copies/mL and 9.00 Log IU/mL (11). Specimen extraction from 200-µL sample input volume was performed with the Qiagen DNA Mini Kit (Qiagen, Hilden, Germany). Altona RealStar BKV ASR was run on the ABI Prism 7500 (Thermo Fisher Scientific, Waltham, MA, USA) per manufacturer’s instructions.

The Roche Cobas BKV assay is run on the Roche Cobas 6800 system, a high-throughput fully automated batch analyzer. The Roche cobas BKV assay is a real-time PCR assay targeting the small t-antigen and VP2 regions of BKV with a sample input volume of 200 µL and a quantitative range between 1.33–8.00 Log IU/mL for plasma and 2.30–8.00 Log IU/mL for urine specimens (12).

### Analytical performance assessment

Alinity m BKV IUO assay linearity was assessed across the range of BKV concentrations from 2.00 to 7.30 Log IU/mL using a commercially available BKV panel in plasma (Exact Diagnostics, Fort Worth, TX, USA). Assay precision was evaluated at two sites by testing a total of 30 replicates of each panel member over 5 days. BKV panels used for linearity and precision studies were stored at −20°C upon receipt and thawed at room temperature and transferred to Alinity m Aliquot tubes prior to testing per the manufacturer’s instructions. Alinity m BKV IUO assay detection was assessed by testing 22 replicates at 50 IU/mL that were prepared by diluting a BKV verification panel member (Exact Diagnostics, Fort Worth, TX, USA) in negative BKV plasma.

Alinity m BKV reproducibility was assessed across two sites evaluating the performance of the assay quality controls [high positive control (HPC), and low positive control (LPC)]. A total of 23 replicates were tested each for HPC and LPC.

### Clinical performance evaluation

A total of 360 de-identified plasma specimens were initially tested fresh with the ELITech BKV LDT. Of the 360 plasma specimens, 128 specimens (35.5%) were tested fresh, and 232 specimens (64.4%) were stored at −70°C between 1 and 34 days prior to testing with Alinity m BKV IUO. An additional 100 de-identified positive plasma specimens previously tested with Altona RealStar BKV assay were procured from iProcess Global Research (Irving, TX, USA); the length of storage in −70°C prior to receipt was not available. Specimens were stored at −70°C upon receipt and aliquoted and stored at −70°C between 8 and 13 days prior to testing with the Alinity m BKV IUO and Roche cobas BKV assays.

A total of 245 urine specimens were initially tested fresh with the ELITech BKV LDT assay. Urine specimens for ELITech BKV LDT testing were prepared by transferring 10 µL of neat urine into 990 µL of a 0.1-mg/mL yeast solution (Invitrogen, Carlsbad, CA, USA, Cat. No. AM7118). A 2.4-mL aliquot of neat urine was transferred and stored in the Alinity m Urine Transport Kit containing 1.2 mL of urine stabilizing solution within 24 hours of collection and stored at 2°C–8°C between 0 and 10 days prior to testing with the Alinity m BKV IUO assay.

### Workflow evaluation

Onboard and processing turnaround times (TATs) of the Alinity m system were evaluated based on the automatic documentation by Alinity m of timepoints for sample loading, sample aspiration, and result reporting. 

### Statistical analysis

All analyses were performed using PC SAS (version 9.4) software (SAS, Cary, NC, USA). Relationships between quantitative values were studied by means of Deming regression. Bland–Altman analysis was performed to evaluate the differences in quantification between the assays.

The following analysis was performed for each instrument and each panel member: the PROC MIXED procedure with the MIVQUE0 option in SAS was used to produce variance components for the model used in the analysis. The point estimates of the means, standard deviations (SD), and % coefficients of variance (CV) were reported. The SD and %CV were estimated for the within-day component, the between-day component, and the between-site component for each instrument and each panel member. All the effects were considered as random for the analyses. Any negative variance components were set to zero for these calculations. Variance components were estimated based on the random effects analysis of variance model: *y* = Mean + Site + Day + Error. The total assay variability was defined as the sum of the within-day (residual error) component, the between-day component, and the between-site component estimates of variability. The following statistics were reported: *N*, mean, within-day SD and %CV, between-day SD and %CV, between-site SD and %CV, and total SD and %CV.

## RESULTS

### Analytical performance

**Fig 1:**
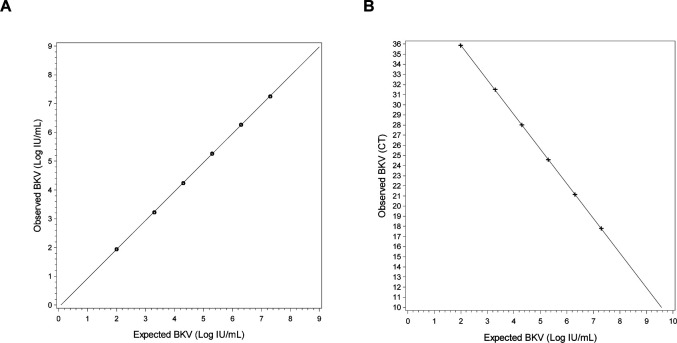
Analytical performance of the Alinity m BKV IUO assay. Linearity was established across the range of BKV concentrations from 2.00 to 7.30 Log IU/mL using commercially available dilution panels in plasma. (**A**) Target concentration versus mean observed concentration. (**B**) Target concentration versus mean observed cycle threshold (CT).

Analytical linearity of the Alinity m BKV IUO assay, using a commercially available panel ranging from 2.00 to 7.30 Log IU/mL, demonstrated a correlation coefficient of 1.000 and inversely correlated with Ct (Fig. 1A and B). Precision analysis demonstrated total CV ranging from 0.8% to 13.0% and total SD of ≤0.25 Log IU/mL for all panel members (Table 1). The Alinity m BKV IUO assay detected 100% (22/22) of replicates at 50 IU/ml.

**TABLE 1:** Precision of the Alinity m BKV IUO assay

Expected concentration (Log IU/mL)	Observed mean concentration (Log IU/mL)	Difference (observed-expected) (Log IU/mL)	Within-day		Between-day		Between-site		Total^*a*^	
			SD	%CV	SD	%CV	SD	%CV	SD	%CV
2.00	1.94	−0.06	0.21	10.6	0.15	7.5	0.00	0.0	0.25	13.0
3.30	3.22	−0.08	0.08	2.5	0.04	1.1	0.03	1.0	0.09	2.9
4.30	4.23	−0.07	0.06	1.4	0.01	0.3	0.00	0.0	0.06	1.4
5.30	5.26	−0.04	0.09	1.7	0.00	0.00	0.02	0.3	0.09	1.7
6.30	6.26	−0.04	0.03	0.5	0.05	0.7	0.00	0.0	0.05	0.9
7.30	7.25	−0.05	0.06	0.8	0.03	0.4	0.00	0.0	0.06	0.8

^*a*^ Total includes within-day, between-day, and between-site components.

Reproducibility of Alinity m BKV IUO testing 23 replicates of HPC and LPC across two sites was characterized by a total %CV of 0.9% and total SD of 0.05 Log IU/mL for HPC and total %CV of 3.7% and total SD of 0.12 Log IU/mL for LPC (Table 2).

**TABLE 2:** Reproducibility

Specimen	Expected concentration (Log IU/mL)	Observed mean concentration (Log IU/mL)	Difference (observed-expected) (Log IU/mL)	Within-day		Between-day		Between-site		Total^*a*^	
				SD	%CV	SD	%CV	SD	%CV	SD	%CV
HPC	6.32	6.21	−0.11	0.01	0.2	0.03	0.5	0.04	0.7	0.05	0.9
LPC	3.22	3.27	0.05	0.09	2.8	0.08	2.4	0.00	0.0	0.12	3.7

^*a*^ Total includes within-day, between-day, and between-site components.

### Clinical performance

A total of 360 remnant de-identified plasma specimens were tested with the ELITech BKV LDT and Alinity m BKV IUO assay. Of the 360 specimens tested with ELITech BKV LDT, DNA was extracted from 190 specimens, and TNA was extracted from 170 specimens using the *m*2000*sp* system.

**Fig 2:**
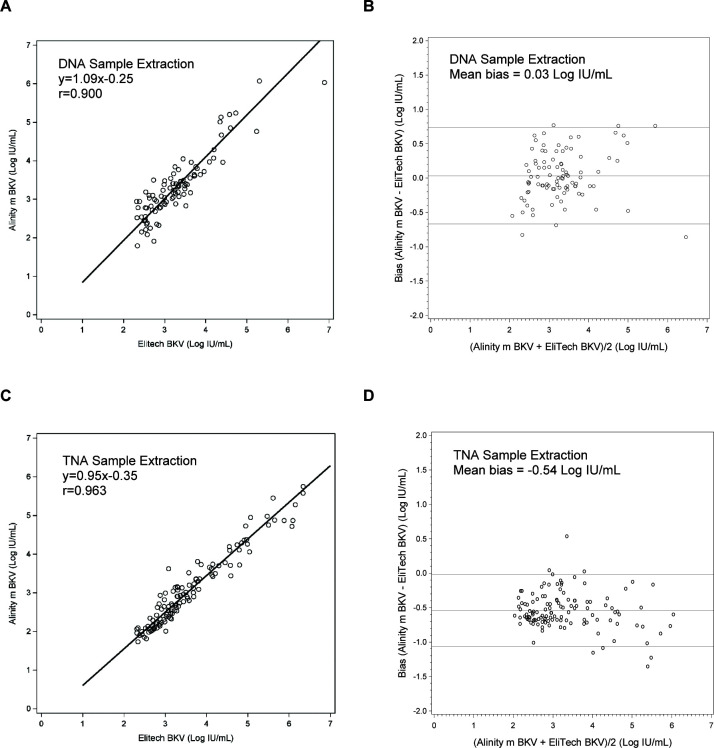
Clinical performance of the Alinity m BKV IUO assay compared to ELITech BKV LDT using DNA and TNA sample extraction protocols for plasma specimens. Deming regression of BKV levels showing correlation between the Alinity m BKV IUO assay and the ELITech BKV LDT using (**A**) DNA sample extraction protocol or (**C**) TNA sample extraction protocol. Bland–Altman analysis showing mean bias between the Alinity m BKV IUO assay and the ELITech BKV LDT using (**B**) DNA sample extraction protocol or (**D**) TNA sample extraction protocol. Solid line indicates mean bias; dotted lines indicate ±1.96 × SD.

Of the 190 specimens in which DNA was extracted for the ELITech BKV LDT assay, overall percent agreement (OPA) with Alinity m BKV IUO was 96.8% with 98.6% positive percent agreement (PPA) and 91.7% negative percent agreement (NPA) with a Cohen’s kappa value of 0.92 representing an almost perfect agreement (Table 3) (13). Ninety-three (*n* = 93) specimens were within the quantitative range of both assays, and the correlation coefficient between the two assays was 0.900 (Deming regression equation, *y* = 1.09 × −0.25; Fig. 2A) with the mean bias of 0.03 Log IU/mL (Bland–Altman analysis, Alinity m BKV–ELITech BKV LDT; Fig. 2B).

**TABLE 3:** Agreement between the Alinity M BKV IUO assay and ELITech BKV LDT using DNA sample extraction protocol as the TOR (*n* = 190 plasma specimens)

	ELITech BKV LDT			
Alinity m BKV IUO	Not detected	Below LLOQ^*a*^	Quantitated	Total
Not detected	44	2	0	46
Below LLOQ^*a*^	4	36	6^*c*^	46
Quantitated	0	11^*b*^	87	98
Total	48	49	93	190

^*a*^ LLOQ used here is the higher LLOQ between Alinity m BKV IUO and ELITech BKV LDT.

^*b*^ 11 specimens detected below LLOQ by ELITech BKV LDT had a range between 2.33 and 3.04 Log IU/mL on Alinity m BKV IUO.

^*c*^ Six specimens detected below LLOQ by Alinity m BKV IUO had a range between 2.34 and 2.74 Log IU/mL on ELITech BKV LDT.

Of the 170 specimens in which TNA was extracted for the ELITech BKV LDT assay, OPA with Alinity m BKV IUO was 97.7% with 100% PPA and 84.0% NPA with a Cohen’s kappa value of 0.90 representing an almost perfect agreement (Table 4). Of the 170 specimens, 123 were quantified by both assays, and the correlation coefficient between the two assays was 0.963 (Deming regression equation, *y* = 0.95 × −0.35; Fig. 2C) and the mean bias of −0.54 Log IU/mL (Bland–Altman analysis, Alinity m BKV–ELITech BKV LDT; Fig. 2D).

**TABLE 4:** Agreement between the Alinity m BKV IUO assay and ELITech BKV LDT assay using TNA sample extraction protocol as the TOR (*n* = 170 plasma specimens)

	ELITech BKV LDT			
Alinity m BKV IUO	Not detected	Below LLOQ	Quantitated	Total
Not detected	21	0	0	21
Below LLOQ^*a*^	4	16	37^*b*^	57
Quantitated	0	0	92	92
Total	25	16	129	170

^*a*^ LLOQ used here is the higher LLOQ between Alinity m BKV IUO and ELITech BKV LDT.

^*b*^ Thirty-seven specimens detected below LLOQ by Alinity m BKV IUO had a range between 2.31 and 3.01 Log IU/mL on ELITech BKV LDT.

**Fig 3:**
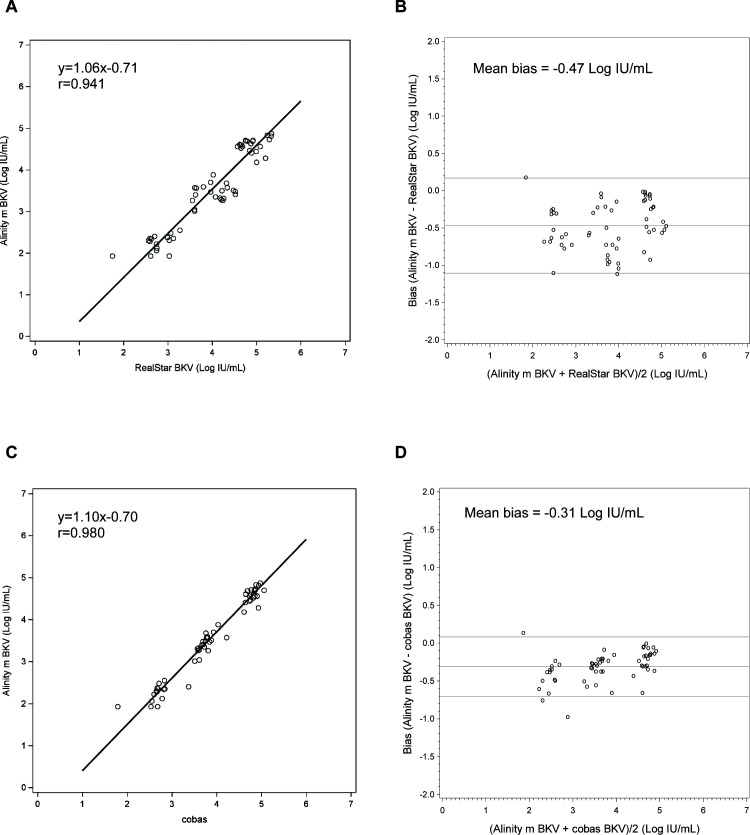
Clinical performance of the Alinity m BKV IUO assay compared to Altona RealStar BKV and Roche cobas BKV assays with plasma specimens. Deming regression of BKV levels showing correlation between the Alinity m BKV IUO assay and the RealStar BKV (**A**) and cobas BKV assay (**C**). Bland–Altman analysis showing mean bias between the Alinity m BKV IUO assay and RealStar BKV (**B**) and cobas BKV assay (**D**). Solid line indicates mean bias; dotted lines indicate ±1.96 × SD.

A total of 100 remnant de-identified positive plasma specimens were tested with the Altona RealStar BKV, cobas BKV, and Alinity m BKV IUO assays. Of the 100 specimens, 59 specimens generated results within the quantitative range of Alinity m BKV IUO and Altona RealStar BKV assays. The coefficient of correlation between the two assays was 0.941 (Deming regression equation, *y* = 1.06 × −0.71; Fig. 3A), and the mean bias was −0.47 Log IU/mL (Bland–Altman analysis, Alinity m BKV–RealStar BKV; Fig. 3B).

Of the 100 specimens, 58 specimens generated results within the quantitative range of Alinity m BKV IUO and Roche cobas BKV assays. The coefficient of correlation between the two assays was 0.980 (Deming regression equation, *y* = 1.10 × −0.70; Fig. 3C), and the mean bias was −0.31 Log IU/mL (Bland–Altman analysis, Alinity m BKV–cobas BKV, Fig. 3D).

**Fig 4:**
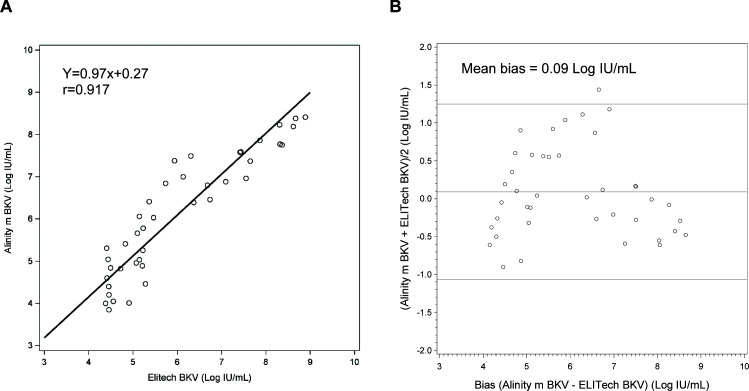
Clinical performance of the Alinity m BKV IUO assay compared to ELITech BKV LDT with urine specimens. Deming regression of (**A**) BKV levels showing correlation between the Alinity m BKV IUO assay and the ELITech BKV LDT(B) Bland–Altman analysis showing mean bias between the Alinity m BKV IUO assay and the ELITech BKV LDT. Solid line indicates mean bias; dotted lines indicate ±1.96 × SD.

A total of 245 urine specimens were tested with the ELITech BKV LDT assay using TNA sample extraction and the Alinity m BKV IUO assay (Table 5). The OPA between the two assays was 84.8% with 96.6% PPA and 81.3% NPA with a Cohen’s kappa value of 0.65 representing a substantial agreement. Of the 245 specimens, 41 specimens were within the quantitative range of both assays. The correlation coefficient was 0.917 (Deming regression equation, *y* = 0.97 × +0.27, Fig. 4A), and the mean bias was 0.09 Log IU/mL (Bland–Altman analysis, Alinity m BKV–ELITech BKV LDT; Fig. 4B).

**TABLE 5:** Agreement between the Alinity m BKV IUO assay and ELITech BKV LDT as the TOR (*n* = 245 urine specimens)

	ELITech BKV LDT				
Alinity m BKV IUO	Not detected	Below LLOQ	Quantitated	Above ULOQ^*b*^	Total
Not detected	152	1	1^*d*^	0	154
Below LLOQ^*a*^	30	9	0	0	39
Quantitated	5^*c*^	3^*d*^	41	0	49
Above ULOQ	0	0	0	3	3
Total	187	13	42	3	245

^*a*^ LLOQ used here is the higher LLOQ between Alinity m BKV IUO and ELITech BKV LDT.

^*b*^ ULOQ used here is the lower ULOQ between Alinity m BKV IUO and ELITech BKV LDT.

^*c*^ Five specimens not detected by ELITech BKV LDT had a range between 4.41 and 4.90 Log IU/mL on Alinity m BKV IUO.

^*d*^ Three specimens detected below LLOQ by ELITech BKV LDT had a range between 4.67 and 5.80 Log IU/mL on Alinity m BKV IUO.

### Workflow analysis

The Alinity m system allowed random continuous loading of BKV specimens side by side with specimens of routine assays that were processed simultaneously on the system. Observed median onboard TAT of clinical specimens from Montefiore Medical Center and MUSC from placement of specimen on the Alinity m system to result reporting for Alinity m BKV was 2 hours and 28 minutes (ranging from 2 hours and 6 minutes to 3 hours and 40 minutes) with median sample processing time of 1 hour and 54 minutes (ranging from 1 hour and 53 minutes to 1 hour 56 minutes).

Abbott *m*2000 system is a batch analyzer that enables laboratories to perform runs of any size (1–96 tests) and then store and reuse activated master mix reagents in subsequent runs. Upon receipt of 100 samples into a laboratory, a single *m*2000 system is able to process 93% of the samples in a standard 8-hour shift, and 7% of the samples are carried over to the next day (14).

## DISCUSSION

This multicenter US study evaluated the performance of the Alinity m BKV IUO assay and demonstrated that the Alinity m BKV IUO assay had SD of ≤0.25 Log IU/mL across the dynamic range (2.00–7.30 Log IU/mL). The 2.00 Log IU/mL panel member included an outlier that was quantitated at 2.99 Log IU/mL. A 100% detection was observed at 50 IU/mL.

Both ELITech BKV LDT study sites utilized the same specimen input volume (500 µL) for specimen extraction on the *m*2000*sp*; however, one site utilized DNA-specific extraction, while the other site utilized TNA-specific extraction. Both extraction methods had strong agreement with the Alinity m BKV IUO assay with an OPA of 96.8% using the DNA extraction protocol and an OPA of 97.7% using the TNA extraction protocol and Cohen’s kappa values of 0.92 and 0.90, respectively. We observed differences in the mean bias between the two extraction methods compared with the Alinity m BKV IUO assay as the DNA extraction protocol resulted in a mean bias of 0.03 Log IU/mL versus the TNA extraction protocol which resulted in a mean bias of −0.54 Log IU/mL. There were 11 specimens that were below the assay lower limit of quantification (LLOQ) by the ELITech BKV LDT but quantitated by Alinity m BKV IUO assay with a range of 2.33–3.04 Log IU/mL. Assay design may contribute to the difference in quantitation as Alinity m BKV IUO is a dual-target assay, whereas ELITech BKV LDT is a single-target assay. Forty-three (*n* = 43) specimens collected from the two sites performing the ELITech BKV LDT testing had results below LLOQ by Alinity m BKV IUO assay and quantifiable results with the ELITech BKV LDTs with a range between 2.31 and 3.01 Log IU/mL. Specimen storage condition and duration prior to testing may contribute to quantitation differences; however, we did not observe a quantitative difference between frozen and fresh specimens when comparing the Alinity m BKV IUO and ELITech BKV LDT; therefore, specimen stability may not be a contributor to the observed bias (data not shown). Differences in precision and calibration strategy may have contributed to the difference in quantitation of the 43 specimens quantitated by ELITech BKV LDT. Alinity m viral load assays utilize an external calibration curve to determine the concentration of an analyte in a patient sample. The analyte signal in a patient sample is compared to a set of samples with a known concentration, and a simple linear regression (*y* = *mx* + *b*) is used to calculate the viral load. This approach typically uses calibrators that are processed as patient specimens through the entire process, allowing for calibration of both extraction and amplification reagents and instruments. Some assays utilize calibrators that are not processed through the extraction; however, this approach carries the risk that difference in recovery or a change in the reagent composition would not be accounted for, potentially leading to differences in quantitation. Another less frequently used strategy is an internal quantitative standard. This application uses a third-order polynomial regression line (*y* = *ax*^3^ + *bx*^2^ + *cx* + *d*) across the linear range with an allowable maximum difference from linearity. Previous studies have suggested that the acceptable allowable difference from linearity for some of the assays was ±0.2 Log10 (15). This allowable difference from linearity and the calibration approach also explains the bias often observed between methodologies, as well as larger imprecision at the low end of the dynamic range where clinical decisions are often being made. Another limitation may be the calibration material that is used for the quantitation of the LDT: how was the material standardized, how was its value assigned, and how frequently the calibration material needed to be run. A limitation of this study is that these two LDTs were performed on different specimens using different instruments and different lots of LDT reagents. These differences in LDT components may contribute to some of the quantitation differences observed in this study. Unfortunately, there was insufficient specimen volume for further testing.

Clinical plasma specimen testing performed comparing Alinity m BKV IUO to Altona RealStar BKV and Roche cobas BKV assays demonstrated good correlation (0.941 and 0.980, respectively) with the mean bias of −0.47 and −0.31 Log IU/mL, respectively. Differences in quantitation may be due to differences in assay design such as specimen extraction and calibration strategy. Location of the primer and probe target region may also contribute to differences in quantitation as Bateman et al. have shown heterogeneity in quantitation of the WHO international standard that includes complex structural variants such as duplication and deletions may affect the ability of primers and probes to detect BKV when targeting these regions (16, 17). Further investigation by Govind et al. (18) demonstrated copy number variation to be dependent on the target region.

Method comparison for urine specimens was performed at one site testing Alinity m BKV IUO and ELITech BKV LDT using TNA extraction. The two methods had a correlation coefficient of 0.917 and mean bias of 0.09 Log IU/mL. Of the 187 specimens in which BKV was not detected by the ELITech BKV LDT assay, 15 were detected below LLOQ by the Alinity m BKV assay, and 20 specimens were quantitated between 1.77 and 5.10 Log IU/mL. Discordant results may be attributed to differences in preanalytical urine preparation as described in Materials and methods. While the LLOQ of the Alinity m BKV IUO assay is the same between plasma and urine specimen types (1.70 Log IU/mL), the LLOQ of ELITech BKV LDT for urine specimens is 4.30 Log IU/mL. The higher LLOQ of ELITech BKV LDT may delay detection of increased BKV viral load due to reactivation. The lower LLOQ of Alinity m BKV IUO enables physicians to observe viral load trends earlier and take action to intervene and modulate immunosuppression sooner.

The median onboard TAT from placement of specimen on the Alinity m system to result reporting was 2 hours and 28 minutes which falls within the range for other Alinity m assays as observed by Obermeier et al. (19) investigating Alinity m TAT in eight sites testing the Alinity m HIV, HBV, HCV, STI, and HPV assays. The sample processing time was shorter than ELITech BKV LDT run on the *m*2000, a batch analyzer with separate sample extraction and amplification step with a total processing time of approximately 7 hours (personal communication) which was within the range observed by Galindo et al. (20) for the Abbott RealTime HIV-1, HBV, HCV, STI, and HPV assays run on *m*2000. The sample processing time of 1 hour and 54 minutes for Alinity m BKV IUO is also shorter than the Roche cobas BKV sample processing time of approximately 3 hours (21). The fully automated, random-access feature of the Alinity m system is predicated on all assays requiring the same amount of time to process a sample, regardless of the assay being tested. This enables multiple assays to be tested concurrently as needed and removes the need to accumulate samples prior to testing on a batch analyzer. The random and continuous access capabilities, as well as the STAT function, which was not tested in this study of Alinity m provide same-day reporting of actionable results.

## References

[1] Borriello M, Ingrosso D, Perna AF, Lombardi A, Maggi P, Altucci L, Caraglia M. 2022. BK virus infection and BK-virus-associated nephropathy in renal transplant recipients. Genes (Basel) 13:1290. doi:10.3390/genes13071290 35886073 PMC9323957

[2] Hirsch HH. 2005. BK virus: opportunity makes a pathogen. Clin Infect Dis 41:354–360. doi:10.1086/431488 16007533

[3] Mbianda C, El-Meanawy A, Sorokin A. 2015. Mechanisms of BK virus infection of renal cells and therapeutic implications. J Clin Virol 71:59–62. doi:10.1016/j.jcv.2015.08.003 26295751 PMC4572911

[4] Ambalathingal GR, Francis RS, Smyth MJ, Smith C, Khanna R. 2017. BK polyomavirus: clinical aspects, immune regulation, and emerging therapies. Clin Microbiol Rev 30:503–528. doi:10.1128/CMR.00074-16 28298471 PMC5355639

[5] Jamboti JS. 2016. BK virus nephropathy in renal transplant recipients. Nephrology (Carlton) 21:647–654. doi:10.1111/nep.12728 26780694

[6] Cesaro S, Dalianis T, Hanssen Rinaldo C, Koskenvuo M, Pegoraro A, Einsele H, Cordonnier C, Hirsch HH, ECIL-6 Group. 2018. ECIL guidelines for the prevention, diagnosis and treatment of BK polyomavirus-associated haemorrhagic cystitis in haematopoietic stem cell transplant recipients. J Antimicrob Chemother 73:12–21. doi:10.1093/jac/dkx324 29190347

[7] Hirsch HH, Randhawa PS, AST Infectious Diseases Community of Practice. 2019. BK polyomavirus in solid organ transplantation-guidelines from the American society of transplantation infectious diseases community of practice. Clin Transplant 33:e13528. doi:10.1111/ctr.13528 30859620

[8] Kant S, Bromberg J, Haas M, Brennan D. 2020. Donor-derived cell-free DNA and the prediction of BK virus-associated nephropathy. Transplant Direct 6:e622. doi:10.1097/TXD.0000000000001061 33134498 PMC7587413

[9] Molecular A. 2022. Alinity m BKV assay IUO clinical brochure

[10] Stepaniants K. 2017. Evaluation of Elitech group’s MGB alert EBV. BK and Adenovirus ASRs on the Abbott M2000sp/Rt Platform, in Clinical Virology Symposium

[11] Diagnostics A. 2018. Realstar BKV PCR kit 1.0. Instructions for Use

[12] Roche, Cobas BKV quantitative nucleic acid test for use on Cobas 6800/8800 systems. 2021. Package Insert

[13] McHugh ML. 2012. Interrater reliability: the kappa statistic. Biochem Med (Zagreb) 22:276–282. 23092060 PMC3900052

[14] Lucic D, Jones S, Wiesneth R, Barry C, Webb E, Belova L, Dolan P, Ho S, Abravaya K, Cloherty G. 2013. Impact of the new Abbott mPLUS feature on clinical laboratory efficiencies of Abbott realtime assays for detection of HIV-1, hepatitis C virus, hepatitis B virus, Chlamydia trachomatis, and Neisseria gonorrhoeae. J Clin Microbiol 51:4050–4054. doi:10.1128/JCM.01672-13 24088850 PMC3838060

[15] Vermehren J, Colucci G, Gohl P, Hamdi N, Abdelaziz AI, Karey U, Thamke D, Zitzer H, Zeuzem S, Sarrazin C. 2011. Development of a second version of the Cobas ampliprep/Cobas taqman hepatitis C virus quantitative test with improved genotype inclusivity. J Clin Microbiol 49:3309–3315. doi:10.1128/JCM.00602-11 21752967 PMC3165622

[16] Bateman AC, Greninger AL, Atienza EE, Limaye AP, Jerome KR, Cook L. 2017. Quantification of BK virus standards by quantitative real-time PCR and droplet digital PCR is confounded by multiple virus populations in the WHO BKV International standard. Clin Chem 63:761–769. doi:10.1373/clinchem.2016.265512 28100494

[17] Govind S, Berry N, Almond N, Morris C. 2017. Harmonization of viral load testing with the first International standard for BK DNA. Clin Chem 63:1902–1903. doi:10.1373/clinchem.2017.279893 29038152

[18] Govind S, Fritzsche M, Jenkins A, Cleveland MH, Vallone PM, Almond N, Morris C, Berry N. 2023. Deep sequencing and molecular characterisation of BK virus and JC virus WHO international reference materials for clinical diagnostic use. Viruses 15:1289. doi:10.3390/v15061289 37376589 PMC10302978

[19] Obermeier M, Pacenti M, Ehret R, Onelia F, Gunson R, Goldstein E, Chevaliez S, Vilas A, Glass A, Maree L, Krügel M, Knechten H, Braun P, Naeth G, Azzato F, Lucic D, Marlowe N, Palm MJ, Pfeifer K, Reinhardt B, Dhein J, Joseph AM, Martínez-García L, Galán J-C. 2020. Improved molecular laboratory productivity by consolidation of testing on the new random-access analyzer alinity m. J Lab Med 44:319–328. doi:10.1515/labmed-2020-0102

[20] Galindo LT, Hristov AD, Gentil LG, Scarpelli L, Santiago J, Levi JE. 2021. Performance evaluation of the fully automated molecular system alinity m in a high-throughput central laboratory. J Clin Virol 137:104786. doi:10.1016/j.jcv.2021.104786 33727012

[21] Song J, Kim S, Kwak E, Park Y. 2023. Evaluating the efficiency of the Cobas 6800 system for BK virus detection in plasma and urine samples. Diagnostics (Basel) 13:17. doi:10.3390/diagnostics13172860 PMC10487002 37685397

